# Genetic architecture of angular leaf spot resistance in cultivated strawberry shaped by epistasis and genotype‐by‐environment interactions

**DOI:** 10.1002/tpg2.70246

**Published:** 2026-05-06

**Authors:** Shai Torgeman, Dominique D. A. Pincot, Marta Bjornson, Randi A. Famula, Paul Skillin, Allison Krill‐Brown, Mitchell J. Feldmann

**Affiliations:** ^1^ Department of Plant Sciences University of California Davis California USA

## Abstract

Angular leaf spot (ALS), caused by *Xanthomonas fragariae*, is a bacterial disease that limits strawberry (*Fragaria × ananassa*) productivity worldwide. Although major resistance loci have been identified in wild *Fragaria* species, their introgression into elite germplasm remains constrained by linkage drag and inconsistent inheritance. To dissect the genetic architecture of ALS resistance, we evaluated a diverse panel of *n* = 241 greenhouse‐tested and *n* = 468 field‐tested strawberry accessions representing elite cultivars, breeding materials, and heirloom varieties of cultivated strawberry (*F. × ananassa*). Plants were evaluated under controlled inoculations and natural field infection using a standardized phenotypic scale and genotyped with 50K single nucleotide polymorphism array. Broad‐sense heritability was moderate (*H* ^2^ = 0.45), whereas narrow‐sense heritability was low (*h* ^2^ = 0.10), reflecting the contribution of nonadditive genetic effects such as epistasis. Genome‐wide association studies (GWAS) identified two loci on chromosomes 1D and 2D, explaining 4%–5% of phenotypic variance, respectively. A segregating F1 population validated the 2D locus, confirmed by co‐localization with the GWAS signal explaining 11.3% of the phenotypic variance. Two‐dimensional genome wide scan in this F1 population revealed a significant epistatic interaction between locus on chromosome 2D and an additional locus on chromosome 6B, collectively explaining 27% of the phenotypic variance. Together, these results demonstrate that nonadditive effects in these populations control ALS resistance. Understanding this complexity provides a foundation for developing elite cultivars with durable resistance, such as UCD Royal Royce. Our findings underscore the need for predictive breeding strategies that capture epistatic and environmental variance to accelerate genetic gain for ALS resistance in strawberry.

## Core Ideas

- Angular leaf spot affects strawberry production globally.
- Resistance loci were discovered on chromosomes 2D, 1D, and 6B.
- Resistance in cultivated strawberry is largely driven by epistasis and genotype‐by‐environmental effects, with specific interactions identified between loci on 2D and 6B.
- Resistance alleles are present in elite germplasm, reducing the need for wild introgression.

## Plain Language Summary

Angular leaf spot is a disease that damages strawberry plants and reduces fruit quality. Finding plants that can resist this disease is important for growers, but most past work has focused on wild strawberry species that are difficult to use in breeding. In this study, we examined many types of cultivated strawberry to see how much natural resistance already exists in varieties that breeders use today. We found that some commercial lines carry useful resistance, even though they do not carry the known resistance gene from wild strawberry. We discovered that resistance comes from several parts of the genome working together, and that these parts interact in ways that make the trait hard to predict. These findings show that future breeding will need to combine multiple resistance genes and test plants in different environments to develop strawberries that stay healthy across growing conditions.

**Abbreviations**
ALS: angular leaf spot
AUDPS: area under the disease progress stairs
CVA: coefficient of additive genetic variance
DPI: days postinoculation
EMM: estimated marginal means
FDR: false discovery rate
FTP: final time point
G × E: genotype‐by‐environment
GH: greenhouse
GWAS: genome‐wide association study
LMM: linear mixed model
LOD: logarithm of odds
MAF: minor allele frequency
MAS: marker‐assisted selection
MV‐GWAS: multivariate genome‐wide association study
OF: open field
PCA: principal component analysis
PVE: phenotypic variance explained
QTL: quantitative trait locus
SNP: single nucleotide polymorphism
UCD: University of California Davis
WEO: Wolfskill Experimental Orchard

## 1 INTRODUCTION

The cultivated octoploid strawberry (*Fragaria × ananassa* Duch.; 2*n* = 8*x* = 56) is a globally popular berry valued for its sweet taste, appealing aroma, attractive appearance, nutritional benefits, and high antioxidant content (Samtani et al., 2019). The cultivated strawberry ranks among the most widely consumed and economically important fruits worldwide. Since 1960, US strawberry production has increased 10‐fold, driven by advances in conventional production systems and the release of improved cultivars (FAOSTAT, https://www.fao.org/faostat/). However, numerous diseases continue to cause substantial economic losses in strawberry production, making resistance traits a top priority for breeding programs.

Angular leaf spot (ALS), caused by the bacterium *Xanthomonas* *fragariae*, was first identified in Minnesota in 1960 (Kennedy & King, 1962). Following its formal classification, the disease rapidly spread across North America and eventually became a global concern, affecting major strawberry‐producing regions in Europe, South America, Africa, and Australia (Gillings et al., 1998; Maas, 1998; Ustun et al., 2007; Zimmermann et al., 2004). This widespread dissemination has been linked to the international exchange of infected planting material, particularly asymptomatic runners harboring latent infections (Jamieson et al., 2013; Roberts, 1996).

The pathogen colonizes host tissues through stomata or wounds and employs a type III secretion system to inject effector proteins that promote colonization and disease development (Kay & Bonas, 2009). Initial symptoms of ALS manifest as small (1–2 mm), water‐soaked lesions bounded by leaf veins, giving them an angular appearance (Kennedy & King, 1962; Maas, 1998). Under transmitted light, these lesions appear translucent and may exude bacterial mucilage under high humidity. Over time, they enlarge, coalesce, become necrotic with a purple border, and produce a tattered leaf appearance. In severe cases, the disease may progress to the crown and vascular tissues, ultimately leading to plant collapse. ALS development is favored by moderate daytime temperatures (∼20°C) and near‐freezing nighttime temperatures, particularly in spring and autumn (Maas, 1998; Roberts, 1996). High humidity, rain, fog, and overhead irrigation further promote its spread (Kaluzna et al., 2019). Yield losses due to ALS range from 8% to 80%, depending on cultivar susceptibility and environmental conditions (Roberts et al., 1997; Epstein, 1966). Although the pathogen does not infect fruit flesh, blemishes on sepals and calyces reduce fruit marketability, leading to lower marketable yield and potential economic loss (Osdaghi, 2024; Roberts et al., 1997).

Consequently, the development of resistant cultivars is regarded as the most practical and sustainable approach for managing ALS, offering both cost‐effectiveness and durability over time. Early screening efforts evaluated diverse Fragaria germplasm, including 81 accessions of F. × ananassa, Fragaria chiloensis, Fragaria virginiana, and Fragaria vesca (Maas et al., 2000). Two resistant genotypes were identified: a native F. virginiana from Minnesota and an *F. virginiana* × *F*. × *ananassa* hybrid (Maas et al., 2000). Follow‐up studies confirmed resistance in breeding lines derived from the *F. virginiana* × *F*. × *ananassa* hybrids named US 4808 and US 4809 (Maas et al., 2002). Initial genetic analyses suggested that ALS resistance is recessive, controlled by two to three loci, with resistant × resistant crosses yielding only ∼30% resistant seedlings (Jamieson et al., 2013; Lewers et al., 2003). Additional resistance sources have since been reported but their use in breeding remains constrained by variable ploidy levels and the presence of undesirable agronomic traits in wild germplasm (Bestfleisch et al., 2015).

Since those initial studies, advances in genetic and genomic technologies (Bassil et al., 2015; Edger et al., 2019; Hardigan et al., 2020; Hardigan, Lorant, et al., 2021) have enabled researchers to dissect the genetic architecture of disease resistance in strawberry with increasing resolution (Mangandi et al., 2017; Nelson et al., 2021; Pincot et al., 2018, 2020).

A major quantitative trait locus (QTL) conferring resistance to ALS, *FaRXf1*, was originally mapped to linkage group 6D (LG6D) using linkage mapping in octoploid strawberry, with marker positions anchored to the diploid *F. vesca* reference genome (Roach et al., 2016). With the subsequent development of high‐quality, haplotype‐phased octoploid strawberry reference genomes and the introduction of *F. ×* *ananassa* based high density single nucleotide polymorphism (SNP) arrays, including the 50K FanaSNP array, subgenome resolution and homoeolog assignment were substantially improved (Bassil et al., 2015; Edger et al., 2019; Hardigan et al., 2020). Under this updated genomic framework, the *FaRXf1* locus was reassigned from LG6D to chromosome 6C (also referred to as chromosome 6–2 in the “Camarosa” genome assembly). This reassignment reflects revised homoeologous chromosome nomenclature and improved genome anchoring rather than the identification of a novel or independent resistance locus (Kim et al., 2025).

Fine‐mapping, bacterial artificial chromosome library screening, and transcriptome profiling of resistant and susceptible genotypes identified FaRGA3 as a strong candidate gene. FaRGA3 encodes a coiled‐coil nucleotide‐binding leucine‐rich repeat protein, with the resistance‐associated allele featuring a distinctive intronic insertion and displaying pathogen‐induced upregulation, consistent with a role in pathogen recognition and defense signaling (Kim et al., 2025). Despite these advances, differences in marker–trait associations across mapping populations and potential strain‐specific differences in pathogen virulence complicate the efficient deployment of ALS resistance in breeding (Kim et al., 2025; Oh et al., 2020; Roach et al., 2016).

Developing ALS‐resistant cultivars with elite horticultural performance is particularly challenging, as the most effective resistance sources to date have come from wild octoploid species such as F. virginiana, which often carry linkage drag and unfavorable agronomic traits. To address this limitation, we evaluated a diverse panel of octoploid *F*. × *ananassa* accessions from the UC Davis strawberry breeding program under both controlled greenhouse (GH) and natural field conditions to assess their resistance to *X. fragariae*. This approach allowed us to identify resistance loci directly within breeding‐relevant *F*. × *ananassa* germplasm, reducing the need for wild introgressions and enabling incorporation of resistant cultivars into breeding pipelines. Our results show that ALS resistance in cultivated strawberry is largely shaped by nonadditive genetic and genotype‐by‐environment (G × E) interactions, emphasizing the complexity of achieving durable resistance through traditional selection.

**Core Ideas**

- Angular leaf spot affects strawberry production globally.
- Resistance loci were discovered on chromosomes 2D, 1D, and 6B.
- Resistance in cultivated strawberry is largely driven by epistasis and genotype‐by‐environmental effects, with specific interactions identified between loci on 2D and 6B.
- Resistance alleles are present in elite germplasm, reducing the need for wild introgression.

## 2 MATERIALS AND METHODS

### 2.1 Plant materials and field experiments

*GH diversity panel*: The population studied in the two GH experiments comprised *n* = 241 *F*. × *ananassa* accessions, including heirloom varieties, breeding lines, and commercial cultivars developed by the University of California strawberry breeding program. Clonally propagated daughter plants were produced from the same set of mother plants grown in low‐elevation nurseries (41 m) in Winters, CA. Although propagation occurred in two seasons, the protocol was standardized across years to minimize developmental differences. Specifically, daughter plants were harvested in October 2024 and February 2025, cold‐stored at −3.5°C for 2–3 weeks, and planted at comparable physiological stages in the Orchard Park GH in Davis, CA.

The GH diversity panel was evaluated using a randomized incomplete block design. Each experiment included approximately *n* = 150 accessions, with an overlap of about *n* = 50 genotypes between years, resulting in a total of *n* = 241 unique accessions. In each experiment, three blocks were used, with a single plant per genotype per block, randomly arranged within the GH. Each plant was tagged on three leaves to record disease severity at multiple time points postinoculation. Plants were grown in 15 cm × 10 cm × 10 cm pots with drip irrigation and standard GH management practices. The experiments were conducted from November 15, 2024, to January 20, 2025, and from March 7, 2025, to May 5, 2025.

*Open‐field (OF) diversity panel*: This panel comprised *n* = 468 *F*. × *ananassa* accessions, including heirloom varieties, breeding lines, and commercial cultivars from the University of California strawberry breeding program with *n = *192 common genotypes shared with the GH experiments. Plants were grown under natural inoculation in OF likely resulting from overhead sprinkler irrigation, in plots of 5–50 plants in a low‐elevation nursery (41 m) at the Wolfskill Experimental Orchard (WEO). The panel was planted in June 2024 and evaluated in January 2025 at the end of the breeding program's maintenance season for this diversity panel, rather than at the end of a commercial production cycle. Phenotyping occurred following multiple rainy periods and nights with low temperatures, conditions that favored disease development and allowed assessment of cumulative disease progression under natural infection pressure.

*F1 population*: This segregating F1 population (*n* = 125) was derived from a cross between a susceptible parent (“19C164P045,” disease severity scored as *𝑦̄* = 3.2 in the GH and *𝑦̄* = 5 in the field) and a resistant parent (“21A137P047,” score of *𝑦̄* = 1 in the field only). Plants were planted in November 2024 and evaluated in March 2025 in the same OF, low‐elevation nursery in Winters, CA that hosted the diversity panel.

### 2.2 Inoculum preparation and inoculation protocol

Two *X. fragariae* strains, *FaP29* and *FaP21*, previously reported to be infectious were provided by Leveau lab from UC Davis on WBN agar plates (Henry et al., 2016). A single colony from each strain was cultured overnight in WBN liquid at 28°C to an OD_600_ of ∼1.0. Cultures were then mixed and diluted to a final concentration of OD_600_ = 0.1, corresponding to approximately 10^7^ CFU/mL (where CFU is colony forming unit) in sterile water. The bacterial suspension was sprayed onto both the abaxial and adaxial surfaces of the leaves and onto the crowns until runoff on the GH diversity panel, in the late afternoon to optimize stomatal receptivity. High humidity conditions (>70%) were maintained postinoculation using a combination of humidifiers and followed by daily misting overhead irrigation for 7 days to enhance inoculum dispersal and sustain high relative humidity within the plant microenvironment (Hildebrand et al., 2005; Roach et al., 2016).

### 2.3 Disease resistance phenotyping

**FIGURE 1:**
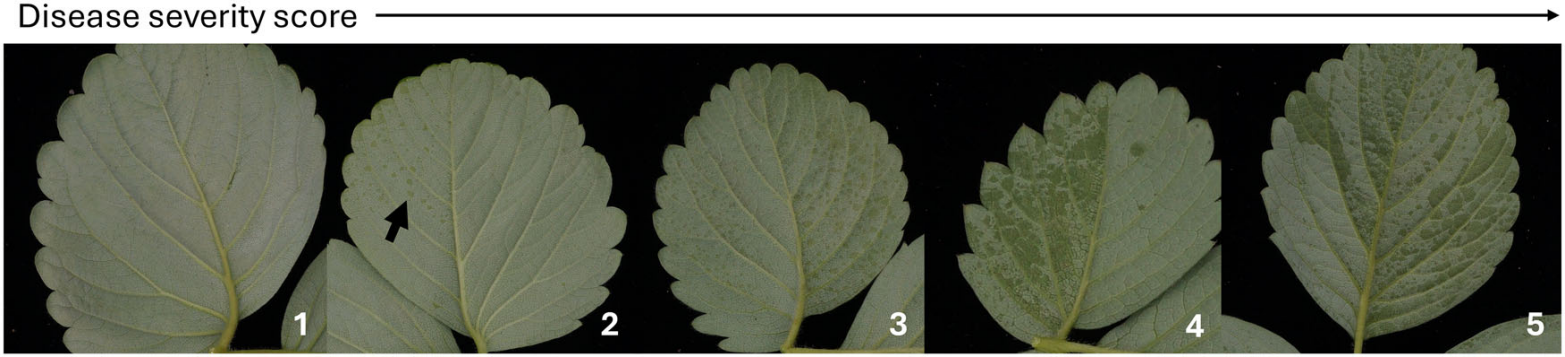
Representative images illustrating the 1–5 disease severity scale used for evaluating angular leaf spot (ALS) symptoms on the abaxial surface of strawberry leaves in greenhouse experiments.

For the GH diversity panel, three fully expanded, healthy leaves per plant were selected at the time of inoculation and permanently tagged. Leaves were chosen from the mid‐canopy to standardize developmental stage and microenvironment and to avoid very young, newly emerging or senescing tissue, which can differ in susceptibility. These leaves were individually scored for water‐soaked lesion severity on a scale between 1 (*most resistant*) and 5 (*most susceptible*) at each time point of 7, 14, 21, and 30 days postinoculation (DPI), following established protocols (Bestfleisch et al., 2015; Hildebrand et al., 2005; Kastelein et al., 2014; Wei et al., 2024). Scores were assigned based on the proportion of symptomatic tissue relative to healthy leaf area, as illustrated in Figure 1. The scoring scale was defined as follows: 1: no visible lesions; 2: lesions covering up to 10% of the leaf surface; 3: lesions covering 10%–20%; 4: extensive dark lesions spreading across the leaf approximately 20%–50%; and 5: lesions covering nearly the entire leaf surface approximately 50%–100%. Photographs of all tagged leaves were taken at the final time point (FTP) (30 DPI) to support documentation and scoring validation. Symptoms had fully progressed and were most extreme among resistant and susceptible checks in the FTP score in each year. We used the package *agricolae*::*audps*() to calculate the area under the disease progress stairs (AUDPS) for each replicate in each year (de Mendiburu, 2021; Simko & Piepho, 2012). We weighted the disease AUDPS by the number of days of evaluation each year so that the minimum value is “1” and the maximum value is “5,” the same scale as for the FTP score. Statistical analyses were applied to both the score and AUDPS traits. For the field diversity panel and the F1 population, disease severity was scored using the same 1–5 ordinal scale. In the field diversity panel, each genotype was represented by a single plot containing 5–50 clonally propagated daughter plants, depending on nursery availability. Disease severity was assessed at the plot level by assigning a single score based on the ratio of necrotic to healthy tissue averaged across all plants within the plot (Figure S1). Thus, individual plants within a plot served as subsamples, whereas the plot constituted the biological unit for statistical analysis. Similarly, in the F1 population, each genotype was represented by a single plant, and disease severity was scored at the plant level following the same approach.

### 2.4 Statistical analyses

For the GH panel analysis, we used the R package *lme4* (Bates et al., 2015) to fit linear mixed models (LMMs) to phenotypic data collected across two experiments. Because the experiment followed an incomplete block design, we modeled experiment and block (nested within experiment) as fixed effects, and genotype‐related sources of variation as random effects. The fitted model expressed as follows:

$$\;y_{ijkr}=\mu +Y_{i}+B{\left(Y\right)}_{j\left(i\right)}+G_{k}+{\left(GY\right)}_{ik}+{\left(GB\left(Y\right)\right)}_{ijk}+\varepsilon_{ijkr}$$

where $y_{ijkr}$ is the disease severity score (averaged across three tagged leaves per plant), $Y_{i}$ is the experiment effect, $B{\left(Y\;\right)}_{j(i)}$ is the block nested within experiment, $G_{k}$ is the genotypic effect, ${\left(GY\;\right)}_{ik}$ is the G × E interaction, ${\left(GB(Y)\;\right)}_{ijk}$ is the genotype × block × experiment interaction, and $\varepsilon_{ijkr}$ is the residual error. Variance components were estimated using restricted maximum likelihood (REML). Broad‐sense heritability (*H* ^2^ = *V*_G_/*V*_P_) on a clone‐mean basis was calculated by *sommer* package in R and was estimated from REML estimates of the accession (*V*_G_), accession × year (*V*_G×Y_), accession × year × block (*V*_G×Y×B_), and residual (*V*_E_) variance components, where *V*_P_ = *V*_G_ + *V*_G×Y_/*y* + *V*_G×Y×B/_ *yb* + *V*_E/_ *ybr*, where *y* is the harmonic mean of number of years (*y *= 1.1), *b* is the harmonic mean of the number of blocks (*b* = 2.4), and *r* is the harmonic mean of number of replications/leaves (*r* = 2.99). *y* was less than two and *b* was less than three due to the nature of the incomplete block designed and because of random missing data, for example, plants that did not survive planting or establishment. The estimated marginal means (EMM) for accessions were estimated using the R package *emmeans* (R. V. Lenth, 2016; R. Lenth & Lenth, 2018) and used as dependent variables (phenotypic observations) for the genome‐wide association study (GWAS). Additionally, we estimated the *H* ^2^_Dominance_ (*𝜎* ^2^_D_ ∕*𝜎* ^2^_P_) and *H* ^2^_Epistasis_ (*𝜎* ^2^_I_ ∕*𝜎* ^2^_P_) components of broad‐sense heritability using the *sommer*::*D.mat()* and *E.mat()* functions on the genotypic matrix, respectively. Genomic‐estimated narrow‐sense heritability (*h* ^2^) was estimated for the GH and field panels using the *rrBLUP::mixed.solve()* function, where *h* ^2^ = *V*_A_/*V*_A_ + *V*_E_, where *V*_A_ is the genomic‐estimated additive genetic variance, and *V*_E_ is the residual variance. Because the field panel dataset included only a single replication per genotype, broad‐sense heritability (*H* ^2^) was not calculated for this panel.

For each significantly associated SNP, the variance explained was estimated from the LMMs described above. Marker variance ($\sigma_{M}^{2}$) was obtained by fitting the SNP as a random effect in the model and extracting the corresponding variance component using REML. To account for unbalanced genotype classes, marker variance estimates were adjusted using the correction described by Feldmann et al. (2021). The proportion of phenotypic variance explained (PVE) by each SNP was then calculated as: PVE = $\sigma_{M}^{2}$ / $\sigma_{P}^{2}$ where $\sigma_{M}^{2}$ represents the variance attributed to the marker effect and $\sigma_{P}^{2}$ denotes the total phenotypic variance.

In addition, we calculated the coefficient of additive genetic variance (CVA), which provides a standardized measure of additive genetic variation across traits and populations: CVA = √(*V*_A_)/*μ*_pop_, where *V*_A_ is the additive genetic variance and *μ*_pop_ is the population mean. Hypergeometric enrichment tests were performed in R using the *phyper* function.

### 2.5 GWAS and multivariate GWAS

SNP genotypes for the GH and field panels were obtained from previous studies (Feldmann et al., 2024; Hardigan, Lorant, et al., 2021; Hardigan, Feldmann, et al., 2021; Pincot et al., 2022). DNA samples of individuals were previously genotyped with Axiom FanaSNP 50k arrays (https://www.thermofisher.com/order/catalog/product/551041) designed by Hardigan et al. (2020). SNP genotypes were curated and filtered for GWAS. The genomic relationship matrix was estimated among individuals using the *A.mat()* function in the R package *rrBLUP* (Endelman, 2011), with a minor allele frequency (MAF) cutoff of 0.05 (*min.MAF *= 0.05), a maximum missing data cutoff of 0.8 (*max.missing* = 0.8), and imputation of missing data (*return.imputed* = TRUE). After data processing, 47,963 SNPs were retained for analyses of the field panel, whereas 47,197 SNPs were retained for analyses of the GH panel. SNP genotypes were recoded as 1, 0, and −1 to match the expectations of the VanRaden method (VanRaden, 2008). To explore the genetic structure of the population, we performed principal component analysis (PCA) using *prcomp()* function of the G matrices. To control for population structure and relatedness in GWAS, we used a Q + K LMM, where Q represents the population structure matrix and K represents the kinship matrix (Yu et al., 2006). We substituted the G matrix for K and incorporated the first three principal components derived from eigenvalue decomposition of the G matrix into the model as covariates. GWAS analyses were conducted using the *GWAS()* function in *rrBLUP*, applied to EMM for accessions between years. Genomic inflation factors (*λ*) after correcting for population structure 1.03 (combined years) and 0.997 (for our field panel analysis), indicating appropriate control of type I error. We used permutation tests to calculate significance thresholds for GWAS hypothesis testing (Gao & Chen, 2011).

We also performed multivariate GWAS (MV‐GWAS) using *GEMMA* v0.98.1 (Zhou & Stephens, 2014), treating the EMMs score and AUDPS as separate but correlated traits. All samples had a call‐rate greater than 90% (<10% missing data), MAF greater than 5% (MAF > 0.05%), and were retained for further analysis. The Affymetrix Axiom Analysis suite and custom R scripts were used to identify and select SNP markers with well‐separated codominant genotypic groups, yielding 19,092 SNP markers from the Axiom array, coded as 0, 1, or 2 (Hardigan et al., 2020). Marker positions were based on the UCD Royal Royce reference genome (Supporting Information S1). The genomic relationship matrix used in *GEMMA* was calculated from these SNPs following the approach of Pincot et al. (2020) and incorporated into MV‐GWAS models to correct for genetic relatedness among individuals. A 5% false discovery rate (FDR)‐corrected threshold was applied to declare significance, following Benjamini and Hochberg (1995).

### 2.6 DNA extraction and genotyping

Leaf samples were collected from the F1 population (*n* = 125) from newly emerged leaves of field‐grown plants into 1.1 mL coin envelopes and freeze‐dried using a Benchtop Pro lyophilizer (VirTis SP Scientific, Stone Bridge, NY). Approximately 0.2 g of dried leaf tissue per sample was transferred into wells of 2.0 mL 96‐well deep‐well plates and ground using stainless steel beads in a Mini 1600 tissue homogenizer (SPEX Sample Prep). Genomic DNA (gDNA) was extracted from powdered leaf tissue using the E‐Z 96 Plant DNA Kit (Omega Bio‐Tek) following the manufacturer's instructions, with minor modifications to improve DNA quality and yield. Specifically, Proteinase K was added to the lysis buffer to a final concentration of 0.2 mg mL^−^^1^, and samples were incubated at 65°C for 45 min. RNA was removed by adding RNase A followed by incubation at room temperature for 5 min prior to centrifugation. Following elution, samples were incubated at 65°C for 5 min to maximize DNA recovery. DNA concentration was quantified using QuantiFluor dye (Promega) on a Synergy HTX microplate reader (BioTek). Genotyping was performed using the Affymetrix 50K SNP Axiom Array (Hardigan et al., 2020) on the GeneTitan HT Microarray platform (Affymetrix). Only high‐quality genomic DNA samples that met both quantity and purity thresholds were processed (e.g., 89% sample call rate, etc.).

### 2.7 Filtering, genetic map construction and QTL analysis

Marker filtering for all samples from the F1 population (*n* = 125) included the removal of loci with a MAF < 0.05, missing rate > 80%, non‐polymorphic markers, and markers showing segregation distortion relative to parental genotypes (*p* > 0.01). After filtering, 17,757 high‐quality SNP markers were retained for downstream analyses.

Genetic distances were calculated using a custom R script, based on the recombination rates between adjacent markers according to their physical positions (Supporting Information S1). Single‐QTL mapping and two‐dimensional genome scans for epistasis detection were performed in R package *qtl* using the EM algorithm (Broman et al., 2003). The QTL confidence interval was defined using a logarithm of odds (LOD) support interval calculated with the *lodint()* function in the R package *qtl*. Single‐marker effects were further analyzed by analysis of variance or Tukey‐Kramer analysis using the JMP Pro 17 software package (SAS Institute). Significance thresholds for each trait were determined using 1000 permutations, generating the empirical distribution of maximum LOD scores. Genome‐wide thresholds were set at the 95th percentile (*p* < 0.05).

## 3 RESULTS AND DISCUSSION

### 3.1 Substantial phenotypic variation was observed for ALS resistance

**FIGURE 2:**
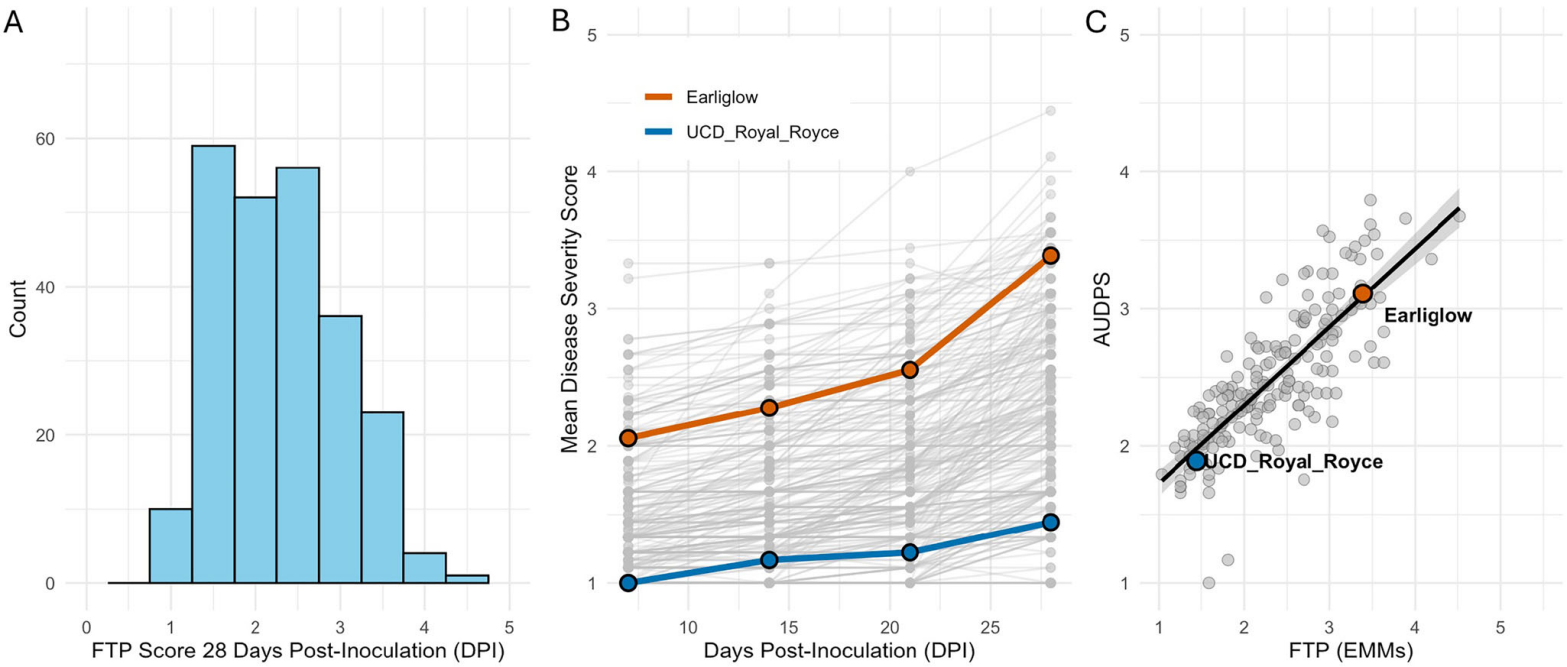
Phenotypic variation and temporal progression of angular leaf spot (ALS) disease severity in the greenhouse (GH) panel of *F*. × *ananassa* accessions inoculated with *Xanthomonas* *fragariae*. (A) Frequency distribution of final disease severity scores (final time point [FTP]) at 28 days postinoculation (DPI) across all genotypes (mean = 2.29), showing a continuous, approximately normal distribution typical of quantitative resistance. (B) Disease progression over time for representative resistant (UCD Royal Royce) and susceptible (Earliglow) cultivars, plotted as mean severity scores at 7, 14, 21, and 28 DPI. Gray lines represent individual genotype trajectories. (C) Correlation between area under the disease progress stairs (AUDPS) and FTP means (FTP estimated marginal means [EMMs]) across the GH panel (*R* = 0.83; *p* = 0.0001).

We observed substantial phenotypic variability for ALS resistance in our GH diversity panel (Figure 2A–C). The between‐year EMMs for the score at the FTP were approximately normally distributed, and the EMMs spanned the entirety of the ordinal scale (Figures 1 **and** 2A). In total, 10,111 phenotypic records across two independent GH experiments were analyzed in this study. We observed that 62% of the strawberry genotypes evaluated in this study were moderately to highly susceptible to ALS, as determined by phenotypic assessments of lesion severity in GH assays (Figure 1). Genotypes were classified into three disease response categories based on FTP scores: highly resistant (1.0 ≤ *𝑦̄* ≤ 1.5), resistant (1.5 < *𝑦̄* ≤ 2.0), and moderately to highly susceptible (2.0 < *𝑦̄* ≤ 5.0). Of the *n* = 241 accessions, 15.7% were classified as highly resistant, 22.4% as resistant, and 61.8% as moderately to highly susceptible. Both University of California Davis (UCD) (*n* = 209) and non‐UCD (*n* = 32) groups exhibited wide variation in ALS resistance, with FTP values ranging from highly resistant (*𝑦̄* < 1.5) to highly susceptible (*𝑦̄* > 4.0). Among the highly resistant accessions (*n* = 38), most (*n* = 29) were UC‐derived cultivars released after 2000; only *n* = 2 were earlier UC selections, and *n* = 7 were non‐UC heirloom lines. Similarly, most resistant accessions (*n* = 47) were UC‐derived (*n* = 36 post‐2000, *n* = 6 pre‐2000, and *n* = 5 non‐UC). The moderately to highly susceptible group (*n* = 145) included many UC materials (*n* = 111 post‐2000, *n* = 14 pre‐2000) as well as non‐UC accessions (*n* = 20). To test whether UCD accessions were over‐represented in any disease‐response class, we performed a hypergeometric test based on their proportion in the full panel (85.9%). The proportions of UCD accessions in the highly resistant, resistant, and susceptible categories did not differ significantly from random expectation (*p *= 0.78, 0.21, and 0.53, respectively). These results indicate that UC‐derived materials are not enriched in any resistance class and that the distribution of resistance reflects the overall composition of the tested population.

Based on our in‐field observations and previous studies, control cultivars performed as expected. The resistant check UCD Royal Royce had a mean disease score of *𝑦̄* = 1.4, while the susceptible check Earliglow averaged *𝑦̄* = 3.4 (Roach et al., 2016; Figure 2B,C). As the disease progressed, the phenotypic distribution shifted toward normality, with an increased proportion of susceptible lines by 28 DPI (Figure 2A,B). These findings were corroborated by AUDPS scores, which showed a strong positive correlation with final disease scores (Figure 2C; *R* = 0.83*; p* = 0.0001). Although extending the experiment to 35 or 42 days would likely shift many resistant genotypes toward susceptibility as lesions expand, we suspect that the relative ranks would not change substantially, given the performance of the check varieties in our study compared to other studies (Kastelein et al., 2014; Roach et al., 2016). We standardized the scoring at 28 DPI because symptom expression and lesion differentiation between genotypes were stable at that point, minimizing confounding from leaf senescence and secondary infection.

### 3.2 ALS resistance is dispersed across genetic backgrounds and has not been under selection in California

**FIGURE 3:**
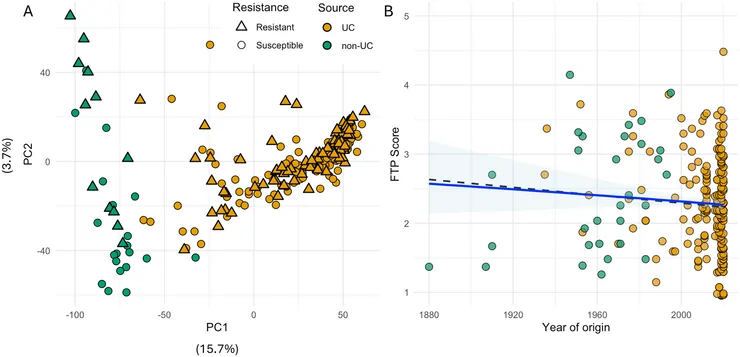
Population structure and temporal trends in angular leaf spot (ALS) resistance across the greenhouse (GH) diversity panel. (A) Principal component analysis of 50K single nucleotide polymorphism (SNP) genotypes showing clear genetic differentiation between UC Davis breeding materials (orange) and non‐UC accessions (green). Resistant and susceptible genotypes are indicated by triangles and circles, respectively. (B) Relationship between cultivar release year and final disease severity (final time point [FTP] score). The solid blue line represents the simple linear regression (*R* = −0.096, p = 0.15), while the dashed line indicates the k‐fold balanced resampling regression (k = 1000; *R* = −0.17, p = 0.01). Shaded areas denote 95% confidence intervals. Both analyses indicate that ALS resistance has not been systematically improved across more than a century of breeding.

PCA of the genetic data for our GH panel revealed clear separation between UCD and non‐UCD germplasm, reflecting their distinct genetic backgrounds (Figure 3A). However, ALS resistance was not associated with specific subpopulations, and resistant and susceptible genotypes were distributed throughout the PCA space, indicating that resistance alleles are not confined to genetic groups. To assess long‐term trends in ALS resistance, we analyzed FTP scores for cultivars with known years of origin, including UCD cultivars (*n* = 209) and non‐UCD germplasm (*n* = 32), spanning over a century of breeding (Figure 3B). Linear regression of FTP on year of origin revealed no significant trend (Figure 3B; *R* = −0.096, *p* = 0.15), suggesting that genetic gains for ALS resistance have been negligible over the last 100 years. Because our panel contains a disproportionately high number of cultivars released after 2018, we performed a *k*‐fold balanced resampling analysis to mitigate bias from recent years dominating the regression. The resampled analyses revealed a slight increase in resistance among newer cultivars (Figure 3B; *R* = −0.17, *p* = 0.01). These finding aligns with the known historical breeding focus of the UC Davis strawberry breeding program, which has prioritized yield, fruit size, and firmness in the long‐term, and more recently resistance to soilborne pathogens such as verticillium wilt, fusarium wilt, and phytophthora crown rot (Feldmann et al., 2024; Pincot et al., 2020; Jiménez et al., 2025; Pincot et al., 2022). By comparison, *X. fragariae* has not posed a significant threat in California strawberry production, and thus, resistance to ALS has not been a consistent breeding target. These results highlight the availability of resistance sources and demonstrate that ALS resistance is already present within commercial cultivars and breeding materials, not only in exotic or wild germplasm. This means that breeders can access resistance alleles directly from adapted backgrounds, avoiding the linkage drag and negative agronomic trade‐offs often associated with introgression from wild species.

### 3.3 ALS resistance is moderately heritable but strongly influenced by epistasis and the environment

Our mixed‐model analysis of the GH panel revealed a moderate broad‐sense heritability (Table 1; *H* ^2^ = 0.45) for disease severity scores, indicating that nearly half of the phenotypic variance is attributable to genetic factors. However, the narrow‐sense heritability was low (Table 1; *h* ^2^ = 0.10), suggesting that additive genetic variance alone explains only a small fraction of the observed variation (Bernardo, 2020; Falconer & Mackay, 1996). Nonadditive genetic sources of variance contributed substantially to the genetic variance, primarily through epistasis (*Epistasis*; *𝜎* ^2^_I_∕*𝜎* ^2^_P_ = 0.23), whereas dominance effects were comparatively minor (*Dominance*; *𝜎* ^2^_D_∕*𝜎* ^2^_P_ = 0.04), underscoring the key role of nonadditive effects in resistance (Holland, 2007; Mackay, 2014). A notable G × E interaction (Table 1; G × E = 16%) was also detected, reflecting substantial variability in clone performance across environments. A low positive correlation between experiments for common genotypes (*n* = 44; *R* = 0.3; *p* = 0.0495) further supports the presence of strong G × E effects. Although plant material and handling were standardized, the two GH trials differed in planting season and environmental conditions, which likely influenced disease development and contributed to genotype‐specific responses. In addition, the limited number of overlapping genotypes reduced the precision of cross‐experiment stability estimates.

**TABLE 1:** Restricted maximum likelihood estimates of broad‐sense heritability (𝐻^2^), narrow‐sense heritability (ℎ^2^), the proportion of the phenotypic variance explained by genotype‐by‐environmental variance (*𝜎* ^2^_G × E_ ∕*𝜎* ^2^_P_), the proportion of the phenotypic variance explained by dominance (*𝜎* ^2^_D_ ∕*𝜎* ^2^_P_), the proportion of the phenotypic variance explained by epistasis (*𝜎* ^2^_I_ ∕*𝜎* ^2^_P_), and the coefficient of additive genetic variation (CVA) for angular leaf spot (ALS) disease severity score.

Trait	Population (*n*)	*H* ^2^	*h* ^2^	*𝜎* ^2^_G × E_ ∕*𝜎* ^2^_P_	*𝜎* ^2^_D_ ∕*𝜎* ^2^_P_	*𝜎* ^2^_I_ ∕*𝜎* ^2^_P_	CVA
Score	GH (241)	0.45	0.10	0.16	0.04	0.23	9.6%
	Field (468)	–	0.20	–	0.19	0.00	18.1%
	Combined (617)	–	0.06	0.18			–
	F1 (125)	–	0.05	–	0.03	0.00	8.9%

Abbreviation: GH, greenhouse.

In parallel with the GH panel, we evaluated a larger diversity field panel (*n* = 468 genotyped accessions) under natural *X. fragariae* infection. Field phenotyping followed the same 1–5 ordinal scale (Figure S1), with average plot‐level scores. The distribution of scores was approximately normal and spanned the entire range, indicating broad phenotypic variation for field resistance.

Of the *n* = 468 accessions evaluated in the field, 8.5% were classified as highly resistant (*𝑦̄* ≤ 1.5), 27.1% as resistant (1.5 < *𝑦̄* ≤ 2.0), and 64.3% as moderately to highly susceptible (*𝑦̄*  > 2.0). Among the highly resistant group (*n* = 40), the vast majority (*n* = 32) were UC‐derived cultivars released after 2000, whereas only two were earlier UC selections and six were non‐UC accessions. Like in our GH panel, the field population also showed no evidence of bias in resistance distribution between UC‐derived and non‐UC accessions. Based on their proportion in the total field panel (84.6% UC), hypergeometric tests indicated that UC‐derived lines were not overrepresented among highly resistant (85%; *p* = 0.39), resistant (83%; *p* = 0.34), or susceptible (81%; *p* = 0.83) accessions. Importantly, long‐term check cultivars included in the panel performed as expected across environments. Two resistant checks, UCD Royal Royce and Victor, consistently exhibited low disease severity, while two susceptible checks, Earliglow and Elsanta, showed high susceptibility, in agreement with prior field‐based evaluations and previous studies (Roach et al., 2016; Gétaz et al., 2020). Specifically, Victor scored *𝑦̄* = 2 in the field and *𝑦̄* = 1.8 in the GH, and UCD Royal Royce scored *𝑦̄* = 2 in the field and *𝑦̄* = 1.4 in the GH. In contrast, Elsanta scored *𝑦̄* = 3 in the field and *𝑦̄* = 3.25 in the GH, and Earliglow scored *𝑦̄* = 4 in the field and *𝑦̄* = 3.4 in the GH. In addition, the highly susceptible genotype Fairland exhibited severe disease symptoms in both environments, scoring *𝑦̄* = 4 in the field and comparably high in the GH.

Notably, UCD Royal Royce exhibited high to moderate resistance under both GH and field conditions despite lacking the *F. virginiana*‐derived *FaRXf1* locus, as characterized by Kim et al. (2025). The observation that UCD Royal Royce does not carry this introgression yet maintains field resistance suggests that additional, independent genomic regions contribute to ALS resistance in cultivated strawberry. These findings highlight the polygenic nature of the trait and reinforce UCD Royal Royce as a valuable source for breeding ALS‐resistant cultivars through marker‐assisted and genomic selection, as well as for studying the genetic mechanisms of ALS resistance that emerged from different sources.

Despite the stable ranking of these check genotypes, the overall correlation between overlapping genotypes evaluated in GH and field trials was low (*n* = 192; *R* = 0.1; *p* = 0.24) and the G × E interaction accounted for 18% of the phenotypic variation (Table 1). This weak correlation likely reflects a combination of strong environmental modulation of disease expression and increased uncertainty associated with field phenotyping (Kaluzna et al., 2019). Field evaluations were conducted under natural infection conditions, with each genotype represented by a single plot per environment and therefore lacking within‐environment biological replication. Although multiple clonally propagated plants were present within each plot and were scored collectively to reduce noise, plot‐level assessments in the field are more variable than GH assays, where inoculum load, temperature, and humidity are controlled. Consequently, low GH–field correlations can arise even when the underlying genetic correlation between true entry means is moderate. The very low phenotypic correlation between GH and OF conditions is also a consequence of attenuation arising from imperfect measurement. For readers less familiar with this phenomenon, the key point is that the correlation between the GH entry means (${\bar{y}}_{\mathrm{GH}}$) and the OF observations ($y_{\mathrm{OF}}$) is reduced because, for single plot‐level observations, LMM analyses cannot fully separate the true entry mean from residual error. Consequently, the OF phenotype contains both signal and measurement noise, which dilutes the covariance between GH and OF phenotypes and mechanically lowers the observed phenotypic correlation.

The preservation of resistance rankings among well‐characterized checks suggests that the GH assay captures biologically meaningful resistance mechanisms, particularly for extreme phenotypes, while variation among the broader diversity panel is more sensitive to environmental heterogeneity and field noise. Notably, this study represents the first time that our germplasm was evaluated under controlled GH and field conditions. Future work may benefit from synchronizing GH and field inoculation protocols, including higher inoculum concentrations, to more closely approximate field disease pressure and improve correspondence between environments.

The narrow‐sense heritability for the OF diversity panel (Table 1; *h* ^2^ = 0.2) was higher than that of the GH panel (*h* ^2^ = 0.1), likely reflecting both the larger sample size and the greater genetic diversity represented in the field experiment. Additionally, phenotypic variance was also higher in the field (*V*_P_ = 0.921) than in the GH (*V*_P_ = 0.474), this difference corresponds to an approximately fourfold increase in additive variance (*V*_A_ = 0.26) compared to (*V*_A_ = 0.06), respectively. The CVA further illustrates this difference, increasing from 9.6% in the GH to 18.1% in the field (Table 1), indicating higher evolvability under natural infection conditions (Houle, 1992). Nonetheless, the overall contribution of additive variance remains low, suggesting that the breeding value for ALS resistance is inherently constrained by low *h* ^2^ and strong environmental influence. Such parameters not only limit the response to selection but also the implementation of genomic prediction for this trait. Similar challenges have been documented in other crops where nonadditive or environmental effects strongly govern disease resistance (Holland et al., 2003; Mackay, 2014). Comparable constraints have been reported for disease resistance traits in other clonally propagated crops, where G × E interactions and nonadditive effects limit additive predictability, such as Phytophthora infestans resistance in potato (Enciso‐Rodriguez et al., 2018). In clonally propagated crops like strawberry, resistant varieties can be directly maintained by vegetative propagation (moderate *H* ^2^). However, progeny from hybrid crosses is likely to segregate for resistance given the low narrow‐sense heritability (Falconer & Mackay, 1996).

This is supported by the observations of Roach et al. (2016) and Kim et al. (2025) who reported that resistant parents derived from *F. virginiana* accessions and their progeny, US4808 and US4809, were intentionally crossed to susceptible breeding lines at each generation, so segregation for ALS resistance was expected; however, susceptible seedlings were still observed among individuals carrying the “resistant” marker haplotype, indicating that additional loci or interactions contribute to resistance. This aligns with the findings of Jamieson et al. (2013), who observed that resistant × resistant crosses yielding only 30% resistant seedlings. Together, these results highlight the challenge of fixing all favorable alleles for a complex, polygenic and environmentally responsive trait that may involve epistatic interactions. They reinforce the need for breeding strategies that integrate marker‐assisted selection (MAS) with large population sizes and multi‐environment testing to more effectively stack resistance alleles in breeding population and commercial strawberry cultivars.

### 3.4 Differences in loci detected by GH and field GWAS reflect environment and population size effects

**FIGURE 4:**
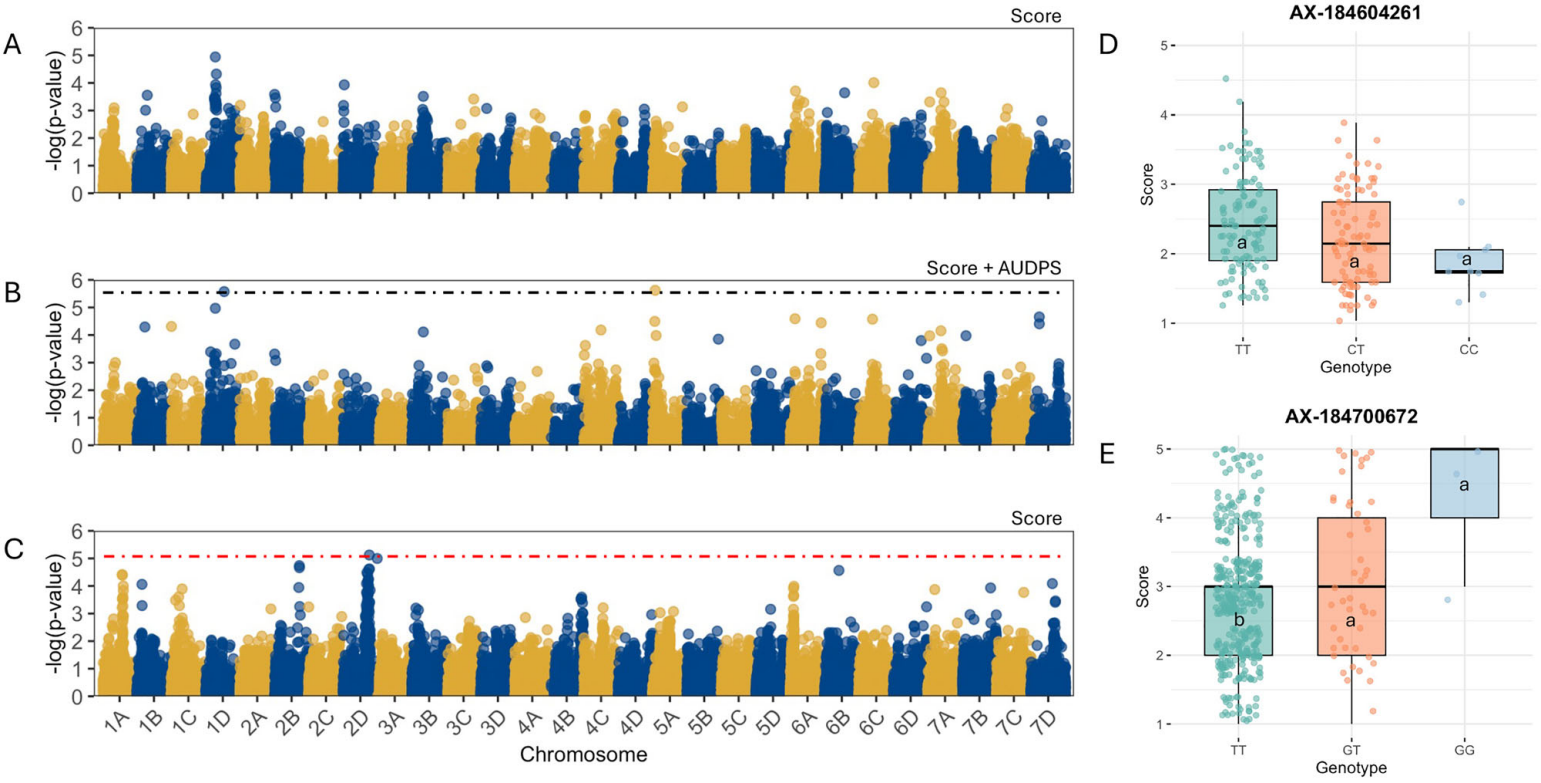
Genome‐wide association analysis of angular leaf spot (ALS) resistance in strawberry. (A–B) Manhattan plots showing marker–trait associations from the greenhouse panel for (A) mean disease severity score and (B) multi‐trait genome‐wide association study (GWAS) combining disease score and area under the disease progress stairs (AUDPS). The black dashed horizontal line indicates the significance threshold determined by false discovery rate (FDR) correction. Significant peak was detected on chromosome 1D. (C) Manhattan plot of field‐based GWAS showing a significant peak on chromosome 2D. The red dashed horizontal line indicates the significance threshold determined by 1000 permutation tests. (D–E) Boxplots showing phenotypic effects of the top single nucleotide polymorphisms (SNPs) AX‐184604261 and AX‐184700672 on chromosomes 1D and 2D, respectively, for disease severity score (estimated marginal means [EMMs]). Different letters indicate groups with statistically significant differences in mean (Tukey Honestly Significant Difference, *p* < 0.05).

To leverage data from both GH and field evaluations, we conducted single‐trait GWAS separately for each environment. In the GH dataset, we additionally performed a MV‐GWAS combining FTP and AUDPS as correlated traits (Zhou & Stephens, 2014). In our GH diversity panel, single‐trait GWAS for FTP did not identify any associations surpassing the permutation‐based significance threshold of *𝑝* = 0.05 or the FDR‐corrected threshold of *𝑝*_FDR_ = 0.05 (Figure 4A). In contrast, MV‐GWAS revealed a single significant SNP on chromosome 1D (AX‐184604261), explaining 4.4% of the phenotypic variance for FTP and 1% for AUDPS (Figure 4B; Table 2). The allele of AX‐184604261 associated with reduced disease severity suggests a potentially additive effect resistance locus; however, the minor genotypic class was represented by only a small number of individuals (Figure 4D; genotypic group “CC,” *n* = 9), limiting our ability to conclusively determine additivity.

**TABLE 2:** Associated single nucleotide polymorphisms (SNPs) and epistatic pair for angular leaf spot (ALS) resistance across populations and environments.

Trait	Population	Chr	Marker ID	Position (Mbp)	PVE%
Score	GH	1D	AX‐184604261	14,163,211	4.4%
	Field	2D	AX‐184700672	27,725,194	5.5%
	F1	2D	AX‐184446554	25,741,239	11.3%
		2D × 6B	AX‐184247661 × AX‐184442490	26,553,233 × 13,220,146	26.8%

Abbreviations: GH, greenhouse; PVE, phenotypic variance explained.

For the field diversity panel, single‐trait GWAS for the FTP identified a significant peak on the south region of chromosome 2D (AX‐184700672), explaining 5.5% of the phenotypic variance (Figure 4C; Table 2). AUDPS was not recorded for the field panel, FTP served as the sole phenotypic measure for GWAS in this dataset. The resistance‐associated allele suggests a potentially additive effect locus, but the third genotypic class is rare, which in turn limits our ability to conclude if the locus is in fact acting additively (Figure 4E; genotypic group “GG,” *n* = 3). The difference in peaks detected between GH and field panels likely reflects both the larger sample size in the field panel, which increases power to detect associations, and the differing environmental conditions and inoculation methods, which can alter trait expression, change the relative contribution of loci to phenotypic variation, and intensify G × E interactions. Further validation of the loci identified here would be strengthened by replicated field trials conducted across multiple years and environments. Replication at the plot level and across seasons would improve the precision of phenotypic estimates, enable more robust partitioning of environmental and G × E effects, and allow assessment of the temporal stability of resistance‐associated alleles under natural infection conditions. Such trials would be particularly valuable for confirming the additive effects and breeding utility of loci detected in single‐year field evaluations, where phenotyping uncertainty and environmental variability are inherently higher.

### 3.5 Validation of GWAS loci in a segregating F1 population

To validate our GWAS findings, we evaluated a segregating F1 family derived from a cross between a susceptible parent “19C164P045” (disease severity scores: *𝑦̄* = 3.2 in the GH and *𝑦̄* = 5 in the field), and a resistant parent “21A137P047” (disease severity score *𝑦̄* = 1 in the field). The susceptible parent “19C164P045” originated from a cross between “16C015P057” and “12C009P605”; with the ALS susceptible variety Fronteras in the pedigree of “12C009P605,” and the resistant “21A137P047” was derived from a cross between “18C449P015” and “18A917P739.”. Notably, UCD Royal Royce appears as a grandparent in both parental lineages through “16C015P057” on the susceptible side and through “18C449P015” on the resistant side making it a common founder of the F1 population, and there is no evidence for a F. virginiana resistance donor in this pedigree (Supporting Information S2). The F1 progeny were evaluated in March 2025 under OF conditions at the WEO. Following several rainfall events, characteristic ALS symptoms developed and disease severity was scored using the same 1–5 ordinal scale described in Figure S1, with average plant‐level scores across all symptomatic leaves. The distribution of severity scores ranged from highly resistant to highly susceptible and appeared normally distributed (Figure S2).

**FIGURE 5:**
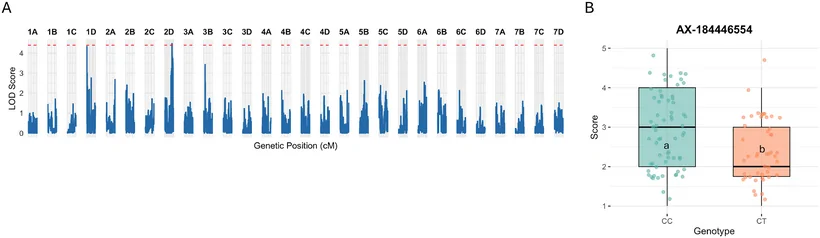
Quantitative trait locus (QTL) mapping of angular leaf spot resistance in a segregating F1 mapping population. (A) Genome‐wide logarithm of odds (LOD) profile showing QTL scan results across the 28 linkage groups of the octoploid genome. The red dashed line indicates the genome‐wide significance threshold (*α* = 0.05) determined by 1000 permutation tests. A significant QTL was identified on chromosome 2D, consistent with the genome‐wide association study (GWAS) peak observed in our field panel. (B) Boxplot showing phenotypic effects of the top marker AX‐184446554 on chromosome 2D for disease severity score (1–5 scale) in the F1 mapping population. The resistant allele is associated with reduced disease severity, validating the QTL identified in the diversity panel. Different letters indicate groups with statistically significant differences in mean (analysis of variance (ANOVA) *t*‐test *p* < 0.05).

QTL analysis revealed a significant peak on chromosome 2D (AX‐184446554; 25,741,239 Mbp) that co‐localized with the GWAS peak found in the field panel (AX‐184700672; 27,725,194 Mbp), suggesting the same causal locus (Figure 5A; Table 2). The difference in peak marker positions reflects the distinct analytical frameworks of linkage mapping and GWAS rather than discordant genetic signals. In the F1 population, QTL positions are determined by recombination breakpoints among informative markers segregating between parents, whereas GWAS peak positions correspond to the single most significantly associated marker in a diverse population. In the F1 mapping population, SNP filtering removed non‐polymorphic markers, markers with segregation distortion, and markers with high missingness, resulting in uneven marker density across the region. Consequently, the peak marker in the linkage analysis represents the most informative segregating SNP within the interval rather than the exact causal position.

The QTL interval spanned from 24,768,387 to 27,878,842 Mbp, overlapping the highest peak detected in our GWAS. Linkage disequilibrium analysis further supported this co‐localization, as markers at the boundaries of the QTL interval were in strong linkage disequilibrium (*r* ^2^ = 0.7). The peak SNP explained 11.3% of the phenotypic variance (Table 2). At this locus, individuals with the heterozygous genotype (“CT”) exhibited reduced susceptibility (*𝑦̄* = 2.1) compared with the susceptible homozygous state (Figure 5B; “CC”; *𝑦̄* = 2.8). Genotype inspection showed that both parents were homozygous at the 1D peak SNP detected in our MV‐GWAS (Table 2), eliminating within‐family segregation and preventing us from evaluating the QTL on chromosome 1D. Overall, these findings validate the chromosome 2D QTL for ALS resistance and highlight its potential utility for MAS in breeding programs.

### 3.6 Epistatic QTL interactions reveal nonadditive genetic control of ALS resistance

**FIGURE 6:**
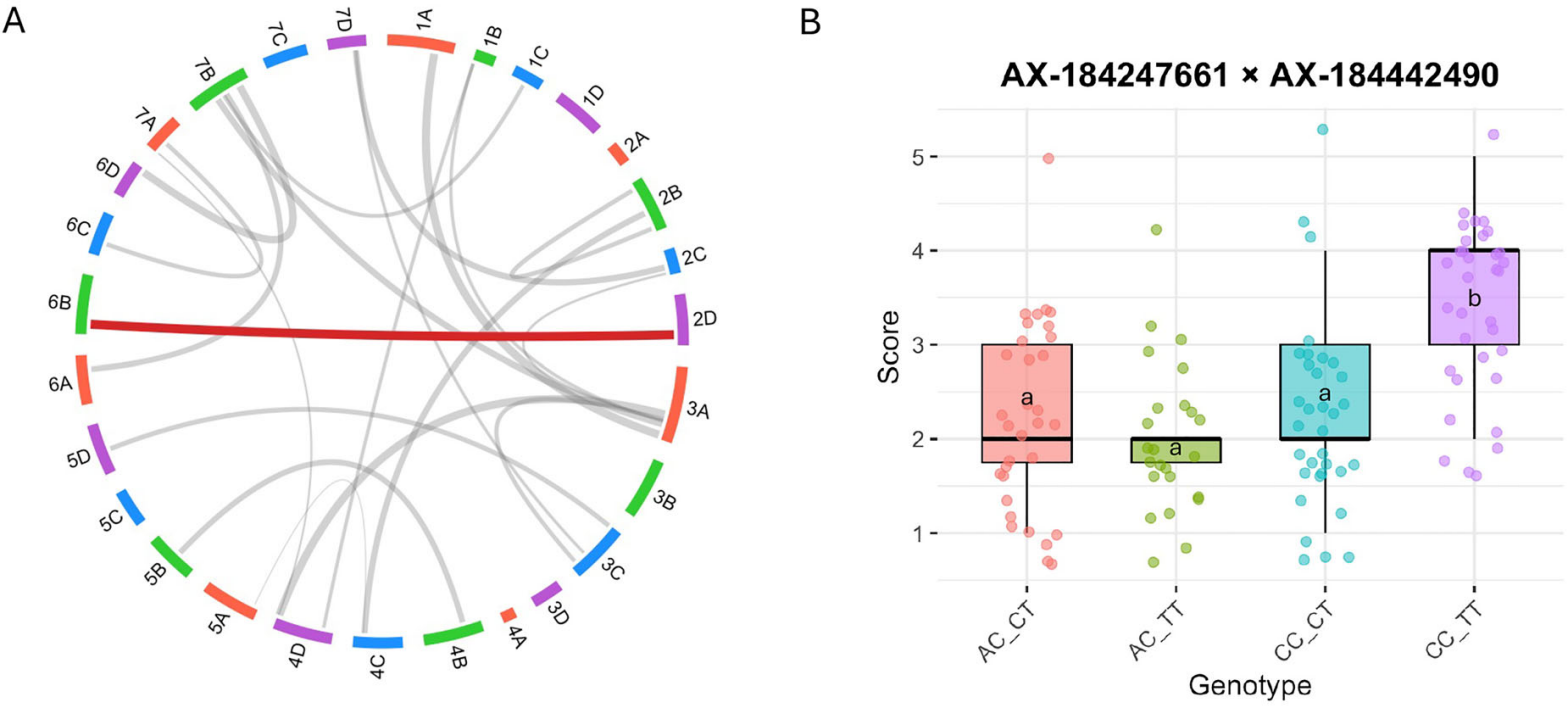
Epistatic interaction underlying angular leaf spot (ALS) resistance in F1 population. (A) Circos plot illustrating significant pairwise epistatic interactions detected across the 28 chromosomes of the octoploid genome. Each gray link represents a significant single nucleotide polymorphism (SNP) × SNP interaction associated with disease severity. The red line highlights the strongest interaction identified between loci on chromosomes 2D and 6B. (B) Boxplot showing the combined allelic effects of the interacting markers AX‐184247661 and AX‐184442490 on chromosomes 2D and 6B respectively for disease severity score (1–5 scale). The double‐homozygous combination ("CC_TT") confers the highest mean disease score, indicating a synergistic (epistatic) effect between these loci that enhances susceptibility. Different letters indicate groups with statistically significant differences in mean (Tukey–Kramer HSD, *p* < 0.05).

Given the substantial proportion of total genetic variance attributable to nonadditive components in our mixed‐model analysis (Table 1), and the large phenotypic variance observed among individuals carrying the favorable allele at the chromosome 2D QTL in the F1 validation population (Figure 5B), we decided to perform a two‐dimensional QTL scan in our F1 population to identify interacting loci underlying ALS resistance. This analysis revealed 20 significant epistatic interactions (Figure 6A; Supporting Information S3). The most significant interaction involved loci on chromosomes 2D and 6B (Supporting Information S3). Notably, the locus on chromosome 2D co‐localized with the QTL interval identified in the single‐locus QTL analysis of the same F1 population (Table 2), and the associated markers were in complete linkage disequilibrium (*r* ^2^ = 1). When the 2D locus (AX‐184247661) combined with the 6B locus (AX‐184442490), the double homozygous genotypic group (“CC_TT”) displayed increased susceptibility to ALS in a more than additive manner (Figure 6B). In contrast, the remaining observed genotype combinations (Figure 6B; “AC_CT,” “AC_TT,” and “CC_CT”) showed comparatively lower susceptibility. Although this genotype combination (“CC_TT”) produced the strongest synergistic effect on susceptibility, the alternative homozygous classes (“CC_CC” and “AA_CC”) cannot be assumed to represent the most resistant background without adequate representation of all nine possible genotypic combinations.

The remaining 19 significant epistatic interactions identified in the scan included all nine possible genotypic combinations across the interacting loci. This more balanced genotype representation may reduce the magnitude of genotype specific effects and could partially explain their comparatively lower statistical significance. Because the chromosome 2D locus was consistently detected in both the single‐locus QTL analysis and the two‐dimensional interaction scan, we focused on the 2D × 6B interaction as the most biologically relevant candidate. Future validation across additional genetic backgrounds will be required to determine the stability and breeding relevance of this interaction.

Consistent with this pedigree context, UCD Royal Royce contains the favorable allele at the chromosome 2D QTL in homozygous state (“CC”) and carries the rare homozygous state at 6B (“CC”), indicating that the 2D × 6B interaction detected here may reflect allelic combinations already present within elite cultivated *F*. × *ananassa* germplasm. Given multiple lines of evidence supporting the resistance of UCD Royal Royce, it will be valuable to investigate whether combining the *F*. *virginiana*–derived *FaRXf1* resistance locus with the 2D × 6B allelic interaction identified in this study could produce even higher and more stable resistance. Further, the UCD Royal Royce phased genome remains one of the highest quality reference genomes for octoploid strawberry research (Hardigan, Feldmann, et al., 2021), making it an ideal candidate for future transcriptomic work to identify genes associated with this novel source of genetic resistance to ALS.

Traditionally, QTL mapping compares trait means across genotypic groups in segregating populations or GWAS, testing markers “one at a time.” This has been foundational for MAS in breeding for disease resistance and crop quality (Collard & Mackill, 2008; Tanksley, 1993). However, despite the ability to score thousands of SNPs simultaneously, such analyses remain largely focused on single markers. Genome‐wide scans for epistatic QTL–QTL interactions can provide better understanding of complex, polygenic traits. These loci may have little or no effect when considered individually but can yield unexpected phenotypic outcomes when combined. Detecting epistasis in segregating populations is inherently challenging for two main reasons: (1) population size, as typical mapping populations of 100–200 individuals are too small to capture all genotype combinations required to detect QTL–QTL interactions, and (2) multiple testing penalty, as scanning all pairwise combinations of loci requires stringent significance thresholds, which in turn reduces power to detect true interactions (Mackay, 2014).

Our GWAS diversity panels were not well‐suited to detect significant epistatic interactions, as each line carries different haplotypes across the genome, making it difficult to identify sufficient individuals sharing the same genotypic combinations to study epistasis. However, in our F1 mapping population, we focused only on co‐segregating markers after filtering based on parental lines' genotypic data, ensuring that each genotypic group was unbiased and represented by an equal number of individuals, allowing epistasis to be detected with greater confidence. We sought to examine the 2D × 6B interaction in the two diversity panels to provide additional supporting evidence for this model of resistance. In our reanalysis, the 2D QTL explained 4.5%–5.5% of the phenotypic variance, and the 2D × 6B interaction explained 4.2%–9% in the GH and OF experiments, respectively. Our detection of a strong epistatic interaction between loci on chromosomes 2D and 6B underscores the role of nonadditive genetic effects in ALS resistance. Notably, this mirrors findings in rice, where combinations of dominant and recessive R genes confer higher levels of resistance through synergistic interactions than any single gene alone (Jiang et al., 2020; Li et al., 2001). Such an example illustrates that alleles with modest or inconsistent single‐locus effects can become essential when combined with complementary loci. In our findings, the 2D × 6B interaction may therefore explain the inconsistent resistance observed in individuals carrying the 2D allele alone, reinforcing the need to consider multi‐locus combinations when designing breeding strategies for durable ALS resistance.

## 4 CONCLUSIONS

Epistasis is well recognized in genetics, as the effect of a gene or QTL often depends on the genetic background, reflecting interactions with unknown loci in the recipient genome (Torgeman & Zamir, 2023). It also plays a central role in hybrid breeding, where hundreds of parental lines are crossed to generate large numbers of experimental F1 hybrids. In California strawberry production, hundreds of new hybrids are tested annually, but only a handful advance to the commercial market. This highlights how only a few specific parental combinations exhibit favorable additive effects and nonadditive interactions necessary for high performance. Developing larger, controlled populations offers a path toward identifying the genomic components underlying epistasis. Such knowledge would enable a more predictive and scientific approach to designing crossing blocks for experimental hybrids, ultimately improving the efficiency of hybrid development for ALS resistance and other agronomic traits.

Our results underscore the importance of considering nonadditive effects and multi‐locus interactions in breeding for disease resistance. From a breeding perspective, ALS resistance is available within elite *F*. × *ananassa* germplasm but has not been a historical target of selection in California. Given its complex, environment‐sensitive nature, strategies that combine MAS, large training populations, and multi‐environment testing will be necessary to capture and fine map dispersed resistance alleles and account for G × E. Overall, our findings indicate that ALS resistance cannot be overcome by single‐gene introgression alone. Instead, predictive breeding approaches that integrate genomic information, epistatic interactions, and field validation will be required to develop cultivars with durable ALS resistance while maintaining agronomic and fruit quality performance.

## AUTHOR CONTRIBUTIONS

**Shai Torgeman**: Conceptualization; data curation; formal analysis; investigation; methodology; project administration; validation; visualization; writing—original draft; writing—review and editing. **Dominique D. A. Pincot**: Conceptualization; data curation; methodology; project administration; writing—review and editing. **Marta Bjornson**: Conceptualization; data curation; writing—review and editing. **Randi A. Famula**: Data curation; project administration; resources; supervision; writing—review and editing. **Paul Skillin**: Data curation; resources; writing—review and editing. **Allison Krill‐Brown**: Conceptualization; data curation; investigation; methodology; project administration. **Mitchell J. Feldmann**: Conceptualization; data curation; formal analysis; funding acquisition; investigation; methodology; project administration; resources; supervision; visualization; writing—review and editing.

## CONFLICT OF INTEREST STATEMENT

The authors declare no conflicts of interest.

## DATA AVIABILITY STATEMENT

The raw phenotypic and genotypic data and the GWAS results generated in this study have been deposited at Zenodo and are freely available (https://doi.org/10.5281/zenodo.17635125).

## Supporting information




**Supplementary Figure S1**. Field symptom scores for angular leaf spot. Representative field images illustrating the 1–5 disease severity scale used in open‐field assessments (1 = no visible symptoms; 2 = few small lesions; 3 = moderate lesions; 4 = extensive, coalescing lesions; 5 = severe, widespread lesions with chlorosis/necrosis).

**Supplementary Figure S2**. Field scoring in the F_1_ validation population. (**A**) Histogram of plant‐level disease severity scores (1–5), showing quantitative segregation with most plants scoring 2–3 and few at the extremes. (**B**) Representative field photo used for scoring calibration, showing characteristic angular leaf spot lesions on a plant with intermediate severity (score 3).

**Supplemental File S1**. The physical positions of 50K Axiom array SNPs corroborated by genetic mapping. SNPs were physically anchored to the ‘Camarosa’ (FaCA1; Edger et al., 2019; https://phytozome‐next.jgi.doe.gov/info/Fxananassa_v1_0_a1) and ‘UCD Royal Royce’ (FaRR1; https://phytozome‐next.jgi.doe.gov/info/FxananassaRoyalRoyce_v1_0) genomes in silico. Chromosomes were numbered using the nomenclature of Hardigan et al. (2020) and cross‐referenced to the nomenclature of Edger et al. (2019). This database includes the physical positions of SNPs identified by BLAST in the ‘Camarosa’ and ‘Royal Royce’ genomes, DNA sequences of the SNP probes, chromosome assignments and physical positions of SNPs corroborated by genetic mapping, and associated information.

**Supplemental File S2**. Detailed pedigree structure for the F1 family derived from the cross between the susceptible parent 19C164P045 and the resistant parent 21A137P047. The pedigree includes all known ancestral lines contributing to each parent, enabling tracking of shared founders, inheritance patterns, and potential sources of allelic variation relevant to ALS resistance

**Supplemental File S3**. Significant epistatic interactions from the two‐dimensional QTL scan.
