# Supplementary material for: Genetic architecture of angular leaf spot resistance in cultivated strawberry shaped by epistasis and genotype‐by‐environment interactions
Source: Plant Genome. 2026 May 6;19(2):e70246. doi: 10.1002/tpg2.70246 (PMC13150400; doi:10.1002/tpg2.70246)
Supplement: Supplementary file 2 [file TPG2-19-e70246-s003.pdf]

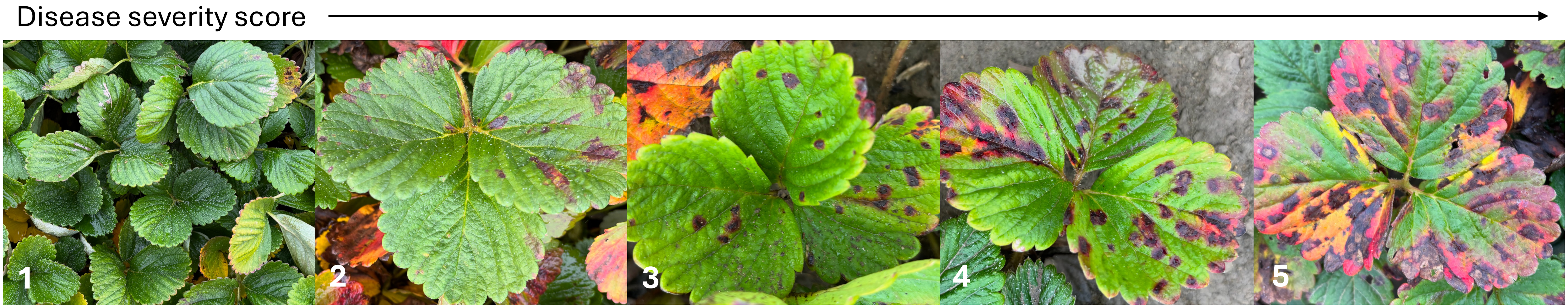
Disease severity score
1
2
3
4
5
