# Supplementary figures and images for: Genetic architecture of angular leaf spot resistance in cultivated strawberry shaped by epistasis and genotype‐by‐environment interactions
Source: Plant Genome. 2026 May 6;19:e70246. doi: 10.1002/tpg2.70246 (PMC13150400; doi:10.1002/tpg2.70246)

Disease severity score 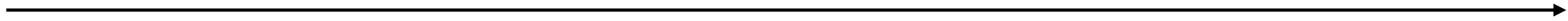

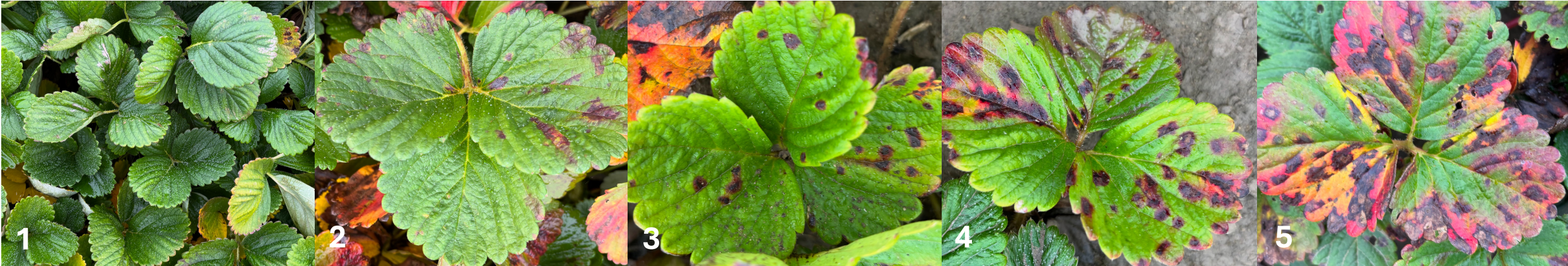

Supplement: Supplementary file 1 — Supplementary Figure S1. Field symptom scores for angular leaf spot. Representative field images illustrating the 1–5 disease severity scale used in open‐field assessments (1 = no visible symptoms; 2 = few small lesions; 3 = moderate lesions; 4 = extensive, coalescing lesions; 5 = severe, widespread lesions with chlorosis/necrosis). [file TPG2-19-e70246-s003.pdf]

A

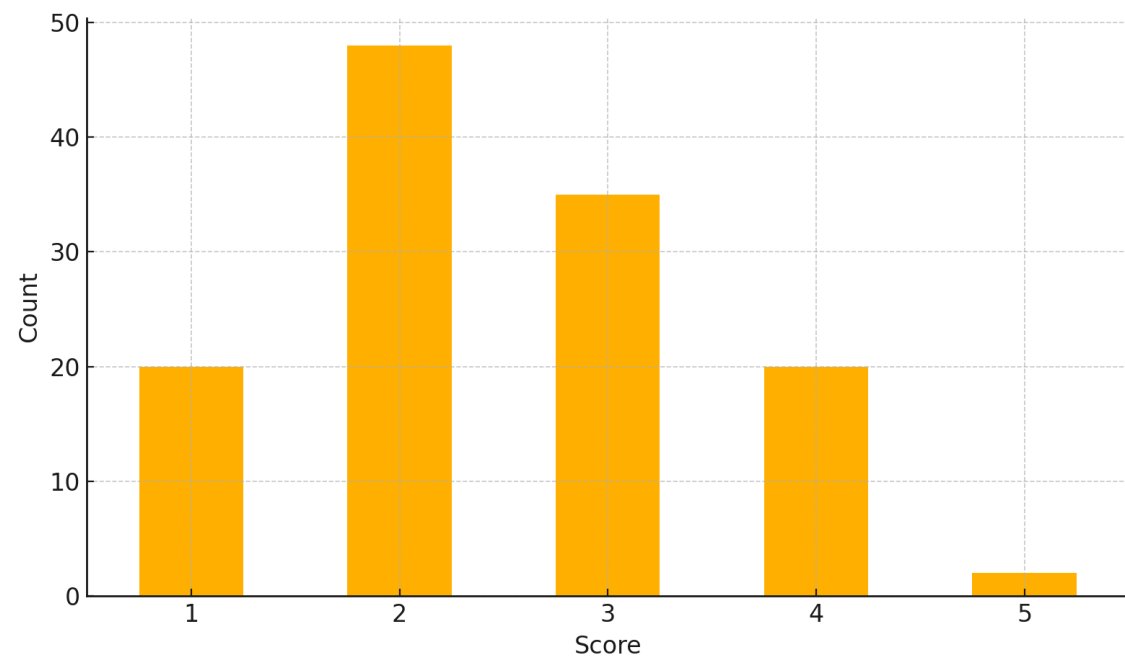

B

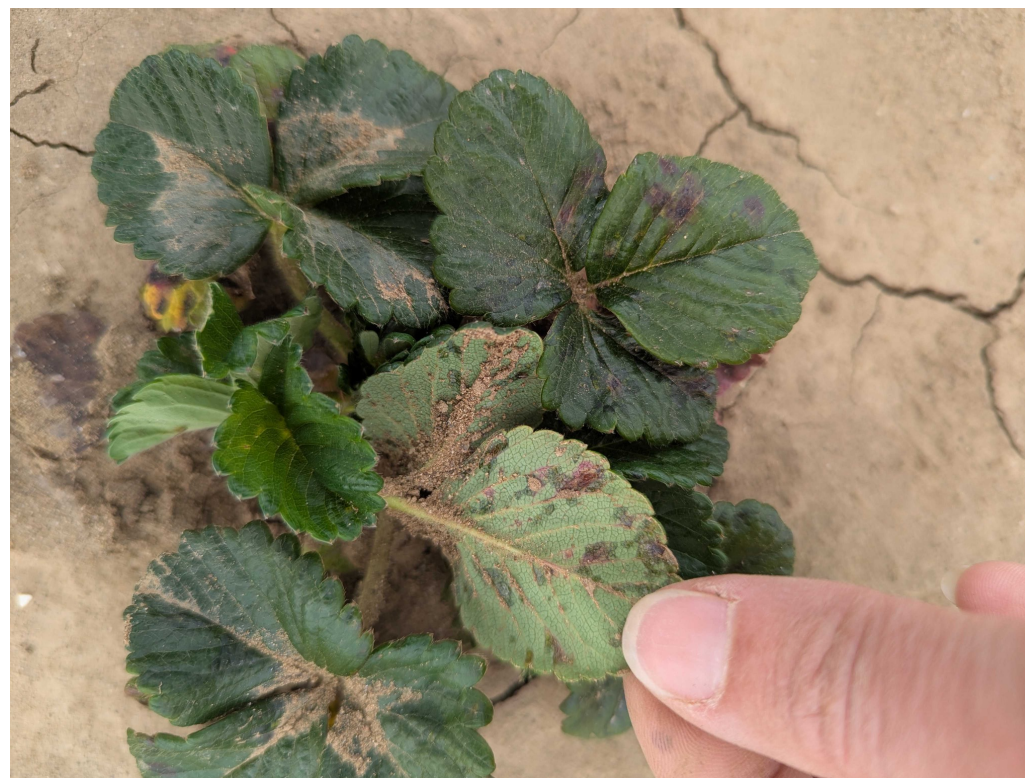

Supplement: Supplementary file 2 — Supplementary Figure S2. Field scoring in the F1 validation population. (A) Histogram of plant‐level disease severity scores (1–5), showing quantitative segregation with most plants scoring 2–3 and few at the extremes. (B) Representative field photo used for scoring calibration, showing characteristic angular leaf spot lesions on a plant with intermediate severity (score 3). [file TPG2-19-e70246-s002.pdf]
